# Consequences of Hatch Phenology on Stages of Fish Recruitment

**DOI:** 10.1371/journal.pone.0164980

**Published:** 2016-10-20

**Authors:** David M. Bogner, Mark A. Kaemingk, Melissa R. Wuellner

**Affiliations:** 1 Kaskaskia Biological Station, Illinois Natural History Survey, Sullivan, IL 61951, United States of America; 2 School of Natural Resources, University of Nebraska-Lincoln, Lincoln, NE 68583, United States of America; 3 Department of Natural Resource Management, South Dakota State University, Brookings, SD 57007, United States of America; University of Hyogo, JAPAN

## Abstract

Little is known about how hatch phenology (e.g., the start, peak, and duration of hatching) could influence subsequent recruitment of freshwater fishes into a population. We used two commonly sympatric fish species that exhibit different hatching phenologies to examine recruitment across multiple life stages. Nine yellow perch (*Perca flavescens*) and bluegill (*Lepomis macrochirus*) annual cohorts were sampled from 2004 through 2013 across larval, age-0, age-1, and age-2 life stages in a Nebraska (U.S.A.) Sandhill lake. Yellow perch hatched earlier in the season and displayed a more truncated hatch duration compared to bluegill. The timing of hatch influenced recruitment dynamics for both species but important hatching metrics were not similar between species across life stages. A longer hatch duration resulted in greater larval yellow perch abundance but greater age-1 bluegill abundance. In contrast, bluegill larval and age-0 abundances were greater during years when hatching duration was shorter and commenced earlier, whereas age-0 yellow perch abundance was greater when hatching occurred earlier. As a result of hatch phenology, yellow perch recruitment variability was minimized sooner (age-0 life stage) than bluegill (age-1 life stage). Collectively, hatch phenology influenced recruitment dynamics across multiple life stages but was unique for each species. Understanding the complexities of when progeny enter an environment and how this influences eventual recruitment into a population will be critical in the face of ongoing climate change.

## Introduction

Understanding recruitment dynamics has long been a challenge in fisheries science [1]. This quest has spanned many decades and environments, resulting in an appreciation for the complexity of factors that shape recruitment among fish populations [2]. Most mortality occurs during the early life stages for fishes (~99%); therefore, understanding events that promote or inhibit recruitment to the next life stage remain important [3,4]. Annual recruitment can be highly variable and affect trophic dynamics, predator-prey relationships, commercial and recreational catch rates, and other population attributes (e.g., growth, mortality; [5–8]). Most previous studies have explored environmental conditions or stock-recruitment relationships to better understand fish recruitment [9]. Yet, fewer studies have examined hatch phenology (e.g., the start, peak, and duration of hatching), collectively, in relation to recruitment across successive life stages.

Reproductive seasons (i.e., when eggs are deposited and hatch) are relatively consistent for fishes and occur within a similar time frame each year (e.g., spring vs. summer). Thus, hatching phenology could explain differences in recruitment dynamics (e.g., consistent vs. erratic, strong vs. weak year classes) among populations. Populations that hatch earlier in the year encounter different environmental conditions compared to populations that hatch later in the year [10]. For example, food availability may differ across time, leading to divergent growth and morality patterns [11,12]. Therefore, reproductive strategies have evolved to account for these temporal changes in resources to ensure viability. The urgency and importance to better understand the role of hatching phenology in shaping recruitment has increased with the onset of climate change [13]. It is unclear how flexible hatch phenology is among populations or how quickly a population can modify this attribute according to environmental conditions [14].

While hatch phenology is relatively consistent on an annual scale (i.e., same season), the timing at which eggs are deposited or larvae hatch within a season is much more variable [15]. This variation could also affect subsequent recruitment [16]. The variability in hatch timing across years within a season may lead to different recruitment outcomes that either promote strong year classes or hinder recruitment [16]. Intra-annual variation in the timing of hatch within a population could be a strategy to optimize current and future environmental conditions for enhanced survival of progeny [17]. The match-mismatch hypothesis illustrates the importance of larvae overlapping adequate prey resources (both spatially and temporally; [18]) but could extend beyond the larval life stage. The timing of available resources or environmental conditions can profoundly alter behaviour, foraging patterns, migrations, predation rates, and many other biological functions [13]. Thus, the ability to modify reproductive timing and output appears to serve as an important dimension within fish recruitment. Individuals within a population may all reproduce simultaneously or spread these reproductive events out over time [19]. Both strategies are accompanied by trade-offs that limit or enhance achieving some fitness or recruitment each year [19].

Yellow perch (*Perca flavescens*) and bluegill (*Lepomis macrochirus*) represent two species that are commonly sympatric but have different annual hatching phenologies and hatch timing within these seasonal spawning events [20,21]. Studying sympatric populations provides a unique opportunity to examine how annual hatch phenology (i.e., spring vs. summer) and hatch timing within a season (e.g., short vs. long reproductive events) can influence early life history and eventual recruitment dynamics. Yellow perch spawn and hatch earlier (spring) and typically channel reproductive efforts into a short, pulsed, and often truncated spawning event (as short as 5–11 days; [22]). In contrast, bluegill spawn and hatch later in the year (early summer) and often spread reproductive efforts across multiple months (87–108 days; [23]), extending into early autumn in some cases.

Our objectives were to 1) explore recruitment dynamics across a series of life stages and identify when recruitment variability diminished for both species; and 2) how the timing of hatch could influence these recruitment dynamics. We predicted that yellow perch recruitment variability would be minimized at an earlier life stage compared to bluegill because most yellow perch recruits experience similar but dynamic, abiotic conditions during spawning and hatching [24]. Therefore, most major mortality events would likely occur during the early life stage (e.g., larval) compared to the juvenile life stage (e.g., age-1). In contrast, weather patterns are generally more stable during summer months when bluegill commence spawning activities, but enter an environment with potentially greater biotic variability [17] resulting in variable but more consistent recruitment across years [25]. Biotic forces such as competition (inter- and intra- specific) with previous spawning fish recruits (e.g, yellow perch [11]), elevated predation rates from newly recruited predators [23], and limited food resources [26] could have greater impact on bluegill than yellow perch populations. Given this high biotic variability operating through time on multiple spawning events and cohorts, we would expect bluegill recruitment to stabilize later in life than yellow perch. These aforementioned differences would uniquely affect how hatch timing could influence recruitment dynamics within these two populations. We hypothesized that hatch phenology will be important for both species but may differ with respect to which attributes are most important (e.g., hatch duration, peak hatch date).

## Methods

All work described herein was approved by the South Dakota State University Institutional Animal Care and Use Committee (Permit Number: 08-A021). Mark Lindvall (refuge manager) and the U.S. Fish and Wildlife Service allowed access to sample Pelican Lake, located within the Valentine National Wildlife Refuge.

### Study area

Pelican Lake is a shallow natural lake with a mean depth of 1.3 m and a surface area of 332 ha located in the Sandhills region of north central Nebraska, U.S.A. inside of the Valentine National Wildlife Refuge (42°31’37”N, 100°40’20”W). The fish community consists of yellow perch, bluegill, largemouth bass (*Micropterus salmoides*), northern pike (*Esox lucius*), black bullhead (*Ameiurus melas*), fathead minnow (*Pimephales promelas*), and common carp (*Cyprinus carpio*).

### Life stage sampling

Larval [total length (TL) < 13 mm] yellow perch and bluegill were sampled every 10 days from April through August or September 2004–2012 during daylight hours. The lake was divided into 16 quadrats; 10 quadrats were randomly chosen and sampled on each occasion [27]. Larval yellow perch and bluegill densities were indexed using a surface trawl with a 0.76-m diameter opening and a 0.1-mm mesh (bar measure) towed in large ellipses for two to five min at an estimated speed of 1.75 m·s^-1^. The volume of water sampled was calculated from a flowmeter (Ocean Test Equipment, Inc., Fort Lauderdale, Florida, USA) mounted in the mouth of the trawl. All fishes collected were preserved in 70% ethanol and transported to the laboratory for identification [28,29] and enumeration. Densities of larvae for each sampling period were calculated as the mean number of fish collected per 100 m^3^ of water filtered. A maximum of 200 individual yellow perch and bluegill per sample were measured for total length (TL; mm).

Sagittal otoliths were removed for aging from three randomly selected yellow perch and bluegill from each of the 10 quadrats sampled on each sampling date (n = 30 per sampling date). Age estimation was completed by two independent readers using a compound microscope; daily ages were averaged if individual reader estimates were within 10% [17,24]. A third experienced reader was consulted if there was no agreement between readers, and the otolith was read in concert until consensus was reached. If all readers failed to reach an agreement, the otolith was removed from the data set (9% and 5% were removed for yellow perch and bluegill, respectively). Larval yellow perch and bluegill hatching distributions for each sampling date were extrapolated to account for all fish sampled [17,24]. Hatch date for individual yellow perch and bluegill was calculated by adding one day and three days to the growth increment count (i.e., circuli) for each species, respectively, to account for swim-up and growth increment deposition differences [17,24]. Hatching distributions were calculated (*H*_*i*_) for each sampling date using the following formula [17]: *H*_*i*_ = (*N*_*i*_/*T*) × *A*, where *i* represents the hatch date, *N* represents the total number of fish aged from hatch date *i*, *T* represents the total number of fish aged, and *A* refers to the total number of fish sampled.

The same yellow perch and bluegill larval cohorts were sampled as age-0 and age-1 using cloverleaf traps in early autumn (August or September) and spring (April or May), respectively [26,30]. Traps were placed in randomly chosen sites in near shore areas of the lake in one to two m of water overnight [31]. Each three-lobed (50 cm in diameter with 41 cm height) cloverleaf trap was constructed of galvanized 6.4-mm bar mesh with three 12.7-mm openings between lobes to accommodate entrance of small yellow perch and bluegill [26]. All fishes were preserved in 90% ethanol before being transported to the laboratory for identification and measured for TL (mm). When a length-frequency histogram did not allow separation between the age classes, a subsample of fish was aged to distinguish age-0 from age-1 fishes in autumn and age-1 from age-2 in spring [31]. These same annual yellow perch and bluegill cohorts were sampled as age-2 using modified fyke nets with 16-mm bar mesh, 1.1- by 1.5-m frames, and 22-m leads in May each year. Fyke nets were set overnight in one to two m of water. All fishes were preserved on ice for transport to the laboratory for TL measurement (mm) and otolith extraction to verify ages as determined by two independent readers. Catch per unit effort (CPUE) was expressed as mean number of fish for ages 0, 1, or 2 of both species per net night.

### Analysis

For this study, we defined recruitment as any fish that survives one life stage and advances to another (e.g., larval to age-0, age-0 to age-1). Thus, recruitment variability was assessed by comparing the relationship of abundances between two consecutive life stages [32,33]. Diminished recruitment variability was evident by a strong relationship between life stages [33], with the earlier life stage defining when recruitment had stabilized. Therefore, this relationship assumes variation in mortality had decreased enough whereby predictions could be made regarding future year class strength [32,34,35]. For example, diminished recruitment variability for age-0 fish would be evident if age-0 abundances were strongly related to age-1 and age-2 life stages but larval abundances were unrelated to these same life stages. In this example, variability in larval abundance was too high to make predictions about year class strength across later life stages (age-0, age-1, age-2). Once we identified the life stage where recruitment variability had diminished, we omitted older life stages in future analyses (i.e., autocorrelated). Pearson’s correlation analysis was used to evaluate variability in recruitment for yellow perch and bluegill by comparing life stage CPUE’s within each species [33,36].

Several hatch timing attributes were calculated each year (2004–2012) from larval otolith age estimates that were hypothesized to influence recruitment of yellow perch and bluegill [22,37–39]. Hatching metrics (calculated using Julian dates) included: earliest-, peak-, and latest- hatch dates; hatch duration; and date at which 50%, the first 30%, and the final 30% of larvae hatched each year (based on total annual larval CPUE). Pearson’s correlation analysis was used to examine multicollinearity among metrics, and only earliest hatch date, peak hatch date, and hatch duration metrics were retained. Earliest hatch date represented the onset of hatching as signaled by the first fish to hatch each year. Peak hatch date corresponded to when the greatest number of larvae hatched. Hatch duration was estimated by subtracting the number of Julian days between the first and last hatch dates.

We used an information theoretic approach (Akaike’s information criterion corrected for smaller sample sizes—AICc; [40]) to examine if hatch attributes were related to different recruitment stages (larval, age-0, age-1) of yellow perch and bluegill across nine annual cohorts (2004–2012). A combination of AICc metrics (differences in AICc scores, model weights) and model fit (R^2^) were used to evaluate model support. A series of regression models (Proc Reg) were developed using yellow perch and bluegill abundances at each recruited life stage (yellow perch = larval, age-0; bluegill = larval, age-0, age-1). The abundance estimates (i.e., CPUE) served as the response variable and hatch attributes (earliest hatch date, peak hatch date, hatch duration) were the independent variables. All variables were tested for normality and log_10_ + 1 transformed as necessary to meet statistical assumptions. All statistical analyses were performed with the Statistical Analysis System (SAS, v.9.4, SAS Institute 2012).

## Results

**Fig 1:**
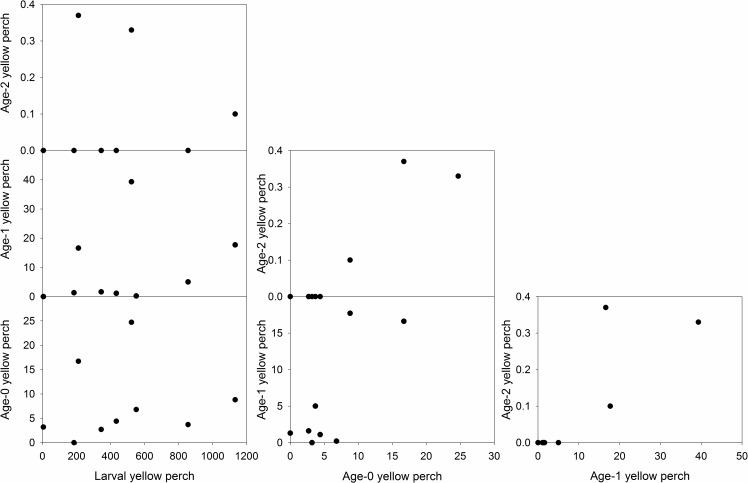
Yellow perch life stage indices and relationships collected from Pelican Lake, Nebraska, USA from 2004 through 2013.

Yellow perch peak larval abundance was not related to age-0 (*r* = 0.30, *p* = 0.44), age-1 (*r* = 0.45, *p* = 0.22), or age-2 (*r* = -0.01, *p* = 0.98) CPUE (Fig 1). However, age-0 yellow perch abundance was related to both age-1 (*r* = 0.70, *p* = 0.04) and age-2 (*r* = 0.82, *p* = 0.01) CPUE. Age-1 and age-2 CPUE’s were also positively related to each other (*r* = 0.82, *p* = 0.01; Fig 1). Therefore, recruitment variability had decreased for yellow perch by age-0 in Pelican Lake.

**Fig 2:**
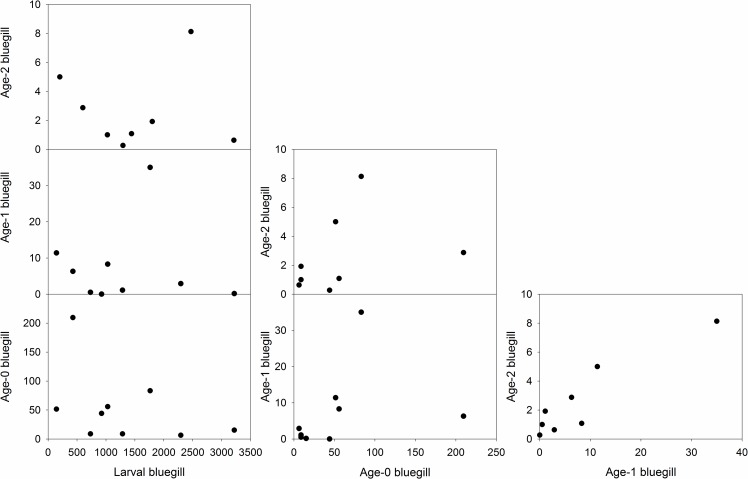
Bluegill life stage indices and relationships collected from Pelican Lake, Nebraska, USA from 2004 through 2013.

Bluegill peak larval abundance was not related to age-0 (*r* = -0.46, *p* = 0.21), age-1 (*r* = -0.25, *p* = 0.51), or age-2 (*r* = -0.15, *p* = 0.72) CPUE (Fig 2). Likewise, age-0 bluegill abundance was not related to age-1 bluegill CPUE (*r* = 0.59, *p* = 0.09) or age-2 bluegill CPUE (*r* = 0.50, *p* = 0.21). However, age-1 CPUE was related to age-2 CPUE (*r* = 0.84, *p* < 0.01; Fig 2). Bluegill recruitment variability had diminished by age-1 in Pelican Lake.

**Fig 3:**
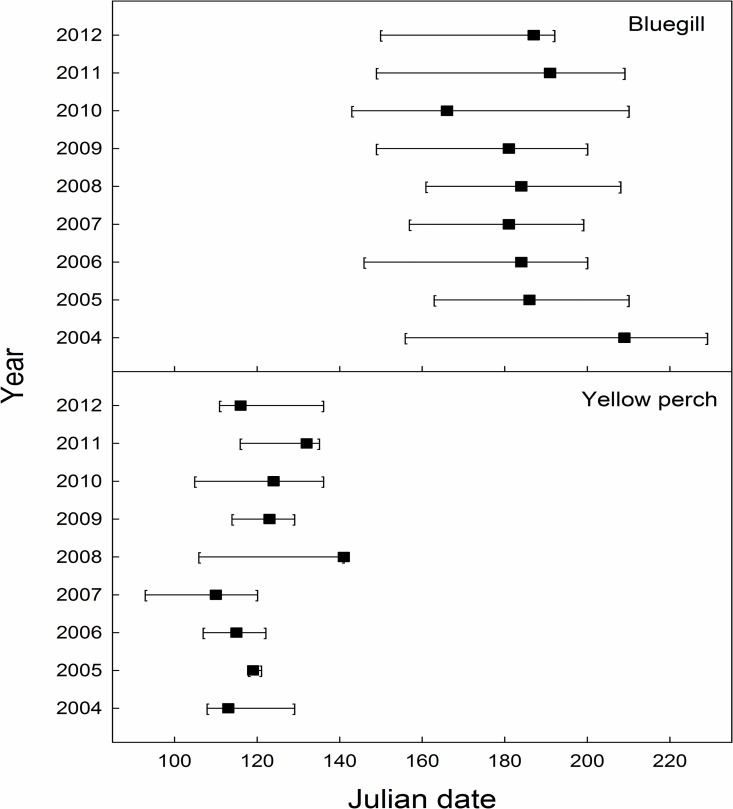
Yellow perch and bluegill earliest (left bracket), latest (right bracket), and peak (box) hatch dates (Julian dates) collected from Pelican Lake, Nebraska, USA from 2004 through 2012. See methods for more details.

**Fig 4:**
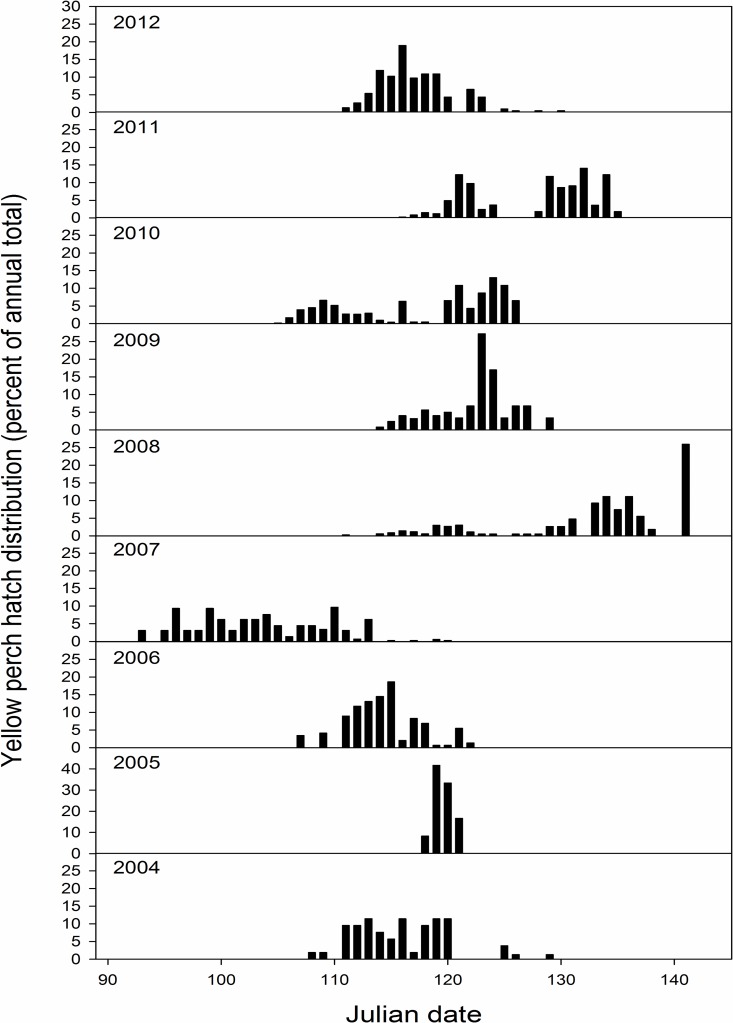
An overview of hatching distributions for larval yellow perch collected from Pelican Lake, Nebraska, USA from 2004 through 2012. Hatching distributions are presented as the percent of annual totals across Julian dates. Earliest and latest hatch dates are not always depicted due to very low representation compared to other dates (please reference Fig 3 for this information).

**Fig 5:**
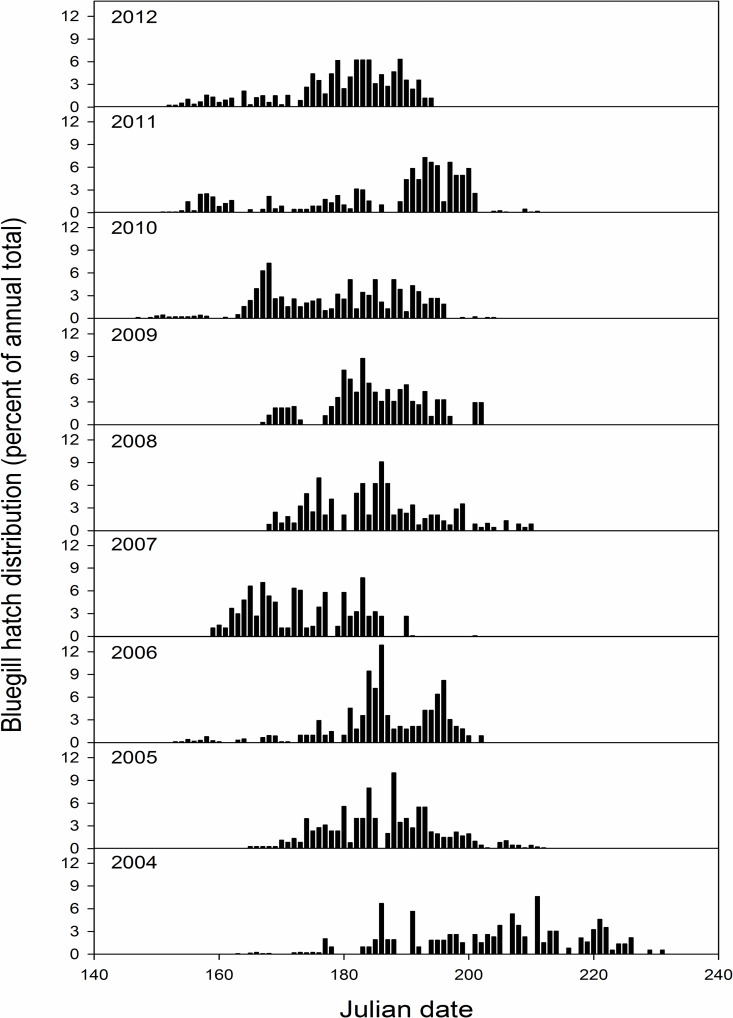
An overview of hatching distributions for larval bluegill collected from Pelican Lake, Nebraska, USA from 2004 through 2012. Hatching distributions are presented as the percent of annual totals across Julian dates. Earliest and latest hatch dates are not always depicted due to very low representation compared to other dates (please reference Fig 3 for this information).

Hatch duration for yellow perch was reduced compared to bluegill, and varied between 3 (2005) and 35 days (2008; Fig 3). Yellow perch earliest hatch date ranged from day 93 (3 April 2007) to 118 (28 April 2005) and peak hatch date varied between day 110 (20 April 2007) and day 141 (21 May 2008; Figs 3 and 4) across years sampled. Alternatively, bluegill exhibited a longer hatch duration ranging from 42 (2007, 2012) to 73 days (2004; Fig 3). Bluegill earliest hatch date ranged from day 143 (23 May 2010) to day 163 (12 June 2005) and peak hatch date ranged from day 166 (15 June 2010) to day 209 (27 July 2004; Figs 3 and 5).

**Fig 6:**
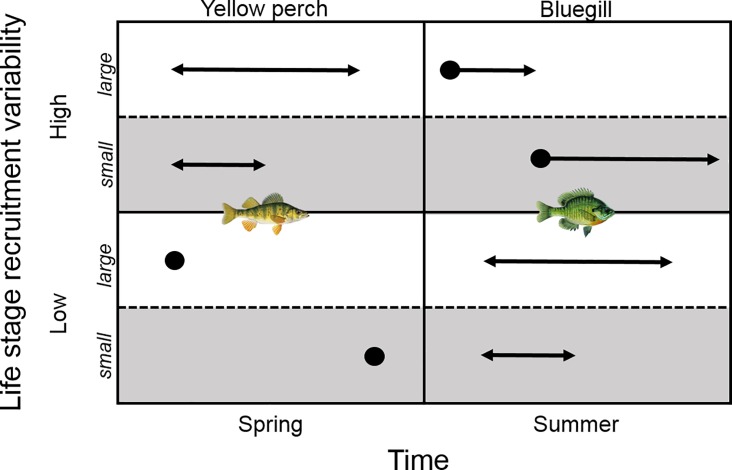
Conceptual model illustrating how hatching phenology affects recruitment dynamics across life stages for yellow perch and bluegill (developed from top AIC_c_ models–Tables 1 and 2). Dots represent the start of hatching and arrow lengths represent duration of hatch. Top panels describe life stages with high recruitment variability and bottom panels describe the life stage where recruitment variability has diminished. White backgrounds indicate large abundances during these life stages whereas gray backgrounds indicate small abundances during these life stages.

The most supported yellow perch larval abundance model included hatch duration, with longer spawning durations associated with greater larval yellow perch abundances (Table 1). Age-0 yellow perch abundance was best explained by earliest hatch date. Earliest hatch dates were negatively related to age-0 yellow perch abundances. Therefore, initiating hatching earlier in the year resulted in higher age-0 yellow perch recruitment (Table 1; Fig 6).

**Table 1:** Akaike’s information criterion rankings to explain larval and age-0 yellow perch abundance (indexed by CPUE) collected from Pelican Lake, Nebraska during 2004–2013. Results include model coefficients for each variable (in parentheses), the number of parameters (K), Akaike information criterion corrected for smaller sample sizes (AICc), differences in AICc (Δ_*i*_), weights (w_*i*_), and the proportion of variance explained (*R*^2^) for each model. Larval and age-0 life stage abundances were log_10_ + 1 transformed.

Model	K	AIC_c_	Δ_*i*_	*w*_*i*_	*R*^2^
*Larval*					
Hatch duration (0.055)	3	3.54	0.00	0.67	0.61
Earliest hatch date (-0.040)	3	6.40	2.86	0.16	0.20
Peak hatch date (0.020)	3	6.91	3.37	0.12	0.09
Earliest hatch date (0.007) + Hatch duration (0.058)	4	10.70	7.16	0.02	0.62
Peak hatch date (0.002) + Hatch duration (0.054)	4	10.73	7.19	0.02	0.62
Earliest hatch date (-0.054) + Peak hatch date (0.033)	4	12.43	8.89	0.01	0.41
Earliest hatch date (0.011) + Peak hatch date (-0.003) + Hatch duration (0.061)	5	22.69	19.15	0.00	0.62
*Age-0*					
Earliest hatch date (-0.031)	3	1.93	0.00	0.48	0.32
Hatch duration (0.010)	3	3.18	1.25	0.26	0.06
Peak hatch date (-0.003)	3	3.40	1.47	0.23	0.01
Earliest hatch date (-0.038), Hatch duration (-0.008)	4	9.01	7.08	0.01	0.34
Earliest hatch date (-0.033), Peak hatch date (0.005)	4	9.06	7.13	0.01	0.33
Peak hatch date (-0.007), Hatch duration (0.013)	4	10.27	8.34	0.01	0.09
Earliest hatch date (-0.062) + Peak hatch date (0.021) + Hatch duration (-0.027)	5	20.36	18.43	0.00	0.44

Larval bluegill abundance was most strongly related to hatch duration, although the model that included peak hatch date was also well supported (Table 2). Hatch duration and peak hatch date were negatively related to larval bluegill abundance; greater larval bluegill abundances occurred during shorter spawning durations and earlier peak hatch dates. The most supported age-0 bluegill abundance model included peak hatch date, but this model explained less variance than other top models. Bluegill abundance at the age-0 life stage was greatest when hatching commenced earlier in the year. Age-1 bluegill abundance was related to hatch duration with longer hatch durations resulting in greater recruitment to this life stage (Table 2; Fig 6).

**Table 2:** Akaike’s information criterion rankings to explain larval, age-0, and age-1 bluegill abundance (indexed by CPUE) collected from Pelican Lake, Nebraska during 2004–2013. Results include model coefficients for each variable (in parentheses), the number of parameters (K), Akaike information criterion corrected for smaller sample sizes (AICc), differences in AICc (Δ_*i*_), weights (w_*i*_), and the proportion of variance explained (*R*^2^) for each model. All life stage abundances and peak hatch dates were log_10_ + 1 transformed.

Model	K	AIC_c_	Δ_*i*_	*w*_*i*_	*R*^2^
*Larval*					
Hatch duration (-0.021)	3	1.78	0.00	0.40	0.32
Peak hatch date (-8.050)	3	2.07	0.30	0.35	0.27
Earliest hatch date (-0.009)	3	3.19	1.42	0.20	0.02
Earliest hatch date (-0.025), Hatch duration (-0.027)	4	7.96	6.18	0.02	0.48
Peak hatch date (-6.250), Hatch duration (-0.017)	4	7.99	6.22	0.02	0.47
Earliest hatch date (0.003), Peak hatch date (-8.459)	4	9.25	7.48	0.01	0.27
Earliest hatch date (-0.016) + Peak hatch date (-3.942) + Hatch duration (-0.023)	5	19.63	17.86	0.00	0.52
*Age-0*					
Earliest hatch date (-0.016)	3	4.42	0.00	0.35	0.12
Hatch duration (0.010)	3	4.71	0.28	0.31	0.05
Peak hatch date (6.565)	3	4.72	0.30	0.30	0.05
Peak hatch date (5.893), Hatch duration (0.006)	4	11.82	7.40	0.01	0.28
Earliest hatch date (-0.012), Hatch duration (0.007)	4	11.54	7.12	0.01	0.07
Earliest hatch date (-0.032), Peak hatch date (9.979)	4	10.80	6.37	0.01	0.14
Earliest hatch date (-0.036) + Peak hatch date (10.990) + Hatch duration (-0.005)	5	22.80	18.37	0.00	0.29
*Age-1*					
Hatch duration (0.024)	3	4.29	0.00	0.38	0.25
Earliest hatch date (-0.029)	3	4.80	0.50	0.29	0.14
Peak hatch date (7.296)	3	4.86	0.56	0.29	0.13
Earliest hatch date (-0.048), Peak hatch date (12.465)	4	10.23	5.94	0.02	0.45
Peak hatch date (5.111), Hatch duration (0.021)	4	11.18	6.88	0.01	0.30
Earliest hatch date (-0.017), Hatch duration (0.020)	4	11.27	6.97	0.01	0.29
Earliest hatch date (-0.041) + Peak hatch date (10.847) + Hatch duration (0.008)	5	22.10	17.80	0.00	0.47

## Discussion

The results of our study highlight that spawning and hatching strategies are very complex and operate on different time scales that ultimately affect the survival of cohorts and recruitment dynamics of a population. As we predicted, recruitment variability was minimized at an earlier life stage for yellow perch that have a spring hatching phenology compared to bluegill that have a summer hatching phenology. The hatching attributes that influenced recruitment also differed between the two species. Hatch timing appears to be important for explaining abundance patterns in the life stages leading up to and at the life stage when recruitment has stabilized. Therefore, it is important to consider the consequence of stage-dependent hatch timing in the recruitment process within a particular spawning and hatching phenology (e.g., spring vs. summer).

While both species have distinct hatching phenologies and strategies, they appear to share some hatching attributes across life stages. A longer hatch duration was related to greater abundance of larval yellow perch and age-1 bluegill. In contrast, initiating hatching earlier corresponded with higher larval bluegill abundance and age-0 yellow perch abundance. Each species shared the importance of hatch duration and initiating hatching earlier but differed with respect to proximate and ultimate effects on recruitment stages. The life stage where recruitment variability becomes diminished is ultimately dependent on when larvae hatch within a given year. Previous studies identified that earlier hatched larval bluegill can experience improved [39] or reduced [23] survival, depending on environmental conditions. It is unclear how these survival patterns ultimately influenced subsequent adult life stages but our study suggests hatch timing has consequences beyond these initial early life stages. Select timing attributes of hatching may promote earlier life stages while others are more critical for later life stages. Or merely, variation in hatch timing is required for successful recruitment. Largemouth bass recruitment has been attributed to multiple hatch-related interconnected critical events during the first year of life [41]. Early-hatched largemouth bass are able to become piscivorous sooner than late-hatched fish, leading to increased access to prey, improved body condition, and higher winter survival [41]. Yellow perch larvae that hatched later in the year exhibited faster growth rates than earlier hatched fish within Pelican Lake, which could influence survival to later life stages [24]. Thus, a series of time-related hatching bottlenecks are responsible for shaping population dynamics.

Factors associated with hatching phenology may be responsible for the recruitment patterns observed. Springtime post-hatch conditions (e.g., water temperatures, precipitation, prey availability) for yellow perch hatching are different than summertime conditions for bluegill. These different biotic and abiotic conditions could explain species-specific differences in recruitment dynamics and why yellow perch recruitment variability is diminished earlier than bluegill. Yellow perch recruitment is most commonly influenced by abiotic or weather related patterns [42] compared to bluegill, which are more susceptible to biotic regulating factors [23,43]. Abiotic or environmental factors are more likely to affect populations over large geographic areas compared to biotic factors that are more spatially isolated or patchy in nature [44]. In the Great Lakes, yellow perch recruitment variability was driven by spring-summer temperatures at a broad scale but subject to differential effects due to biotic factors at the local scale [45]. The protracted spawning and hatching strategy in bluegill may cause a delay in recruitment because multiple cohorts are experiencing unique spatially- and time- dependent biotic and abiotic conditions [17,27].

The interaction of hatching phenology and the reproductive allocation of progeny within a single year has resulted in reproductive tradeoffs that ultimately influence recruitment dynamics. Yellow perch are more likely to have weak year classes or recruitment failure within a given year [46]. Yellow perch place all their eggs in one basket, theoretically speaking, which can result in erratic (strong, weak, or failure; [46]) recruitment patterns, but recruitment variation appears to be more quickly minimized in comparison to bluegill. External factors shape recruitment for yellow perch within the first year by promoting or limiting the survival of a single cohort that hatched within a truncated time frame [22,24]. Alternatively, the bet-hedging (or opportunistic) bluegill strategy ensures at least some reproductive effort will formulate into recruitment [19]. Thus, protracted spawning and subsequent hatching for bluegill is a reproductive strategy that is critically linked to successful recruitment. The recruitment process is therefore extended across multiple life stages and cohorts before mortality has stabilized. Each of these adult populations therefore resembles the combination of multiple aspects of hatching phenology.

Our results suggest that bluegill populations may adapt better to a changing climate compared to yellow perch populations, at least from a hatching phenology perspective. The opportunistic and plastic spawning attributes of bluegill may help them to be more resilient against changes in environmental conditions [24]. Earlier and more dynamic spring conditions [14] may only exacerbate the already erratic recruitment patterns observed in yellow perch populations [46]. Bluegill can modify spawning efforts according to changes in both biotic and abiotic factors [24]. Other species have demonstrated the ability to shift reproductive timing to match changes in local environmental conditions [47,48], even among migratory species where timing is more complex and critical [49]. However, such adaptations have not been demonstrated for yellow perch that are more prone to recruitment failures caused by adverse abiotic conditions. A better understanding of the hatching phenology dimension for fishes will be required to predict how populations will respond to long-term changes in climate [50]. Furthermore, inter- and intra-annual changes in hatch phenology will have consequences on other species and could result in ecosystem level impacts [51,52] We encourage other studies to collectively examine how hatching phenology shapes recruitment dynamics, as this will depend on species, environmental conditions, and geographic location. These studies should also incorporate the interdependency of multiple life stages and evaluate them within an ecosystem context [41,53].

Our study emphasizes the need to collectively incorporate annual hatching phenology and the timing of hatch within a season to address stage-dependent recruitment dynamics in fishes. Environmental conditions experienced at earlier life stages can shape future fitness of individuals at later life stages [54]. Understanding these life stage specific patterns related to the timing of hatch will reveal potential challenges these populations encounter to maximize fitness [41]. The same hatch timing characteristics may not equally promote growth and survival for all life stages but are coupled and interconnected to shape adult population structure and dynamics. Provisioning eggs and larvae to enter an environment that is both currently conducive for survival and equips them for future life stages and conditions is critical [55]. Understanding the mechanisms behind these latent interactions involving hatch will be challenging. However, identifying which populations have the ability to adjust the timing of reproduction will be necessary if we want to evaluate long-term population viability, especially in the midst of a changing climate [50]. The capacity to adjust the timing of reproduction and subsequent hatching attributes could become an even more important dimension for populations that experience high mortality during the early life stages [17,56].

## Supporting Information

S1 Table Catch per unit effort for yellow perch and bluegill year classes (2004–2012) sampled across life stages (larval, age-0, age-1, age-2) from Pelican Lake, Nebraska, USA from 2004 through 2013. No samples (NS) were collected for either species during 2014 to estimate age-2 abundances for the 2012-year class.
(DOCX)
