# Supplementary material for: Consequences of Hatch Phenology on Stages of Fish Recruitment
Source: PLoS One. 2016 Oct 20;11(10):e0164980. doi: 10.1371/journal.pone.0164980 (PMC5072656; doi:10.1371/journal.pone.0164980)
Supplement: S1 Table — No samples (NS) were collected for either species during 2014 to estimate age-2 abundances for the 2012-year class. (DOCX) [file pone.0164980.s001.docx]

**S1 Table**. Catch per unit effort for yellow perch and bluegill year classes (2004-2012) sampled across life stages (larval, age-0, age-1, age-2) from Pelican Lake, Nebraska, USA from 2004 through 2013. No samples (NS) were collected for either species during 2014 to estimate age-2 abundances for the 2012-year class.

| **Species and year class** | **Larval**  **(#/100 m^3^)** | **Age-0**  **(#/net night)** | **Age-1**  **(#/net night)** | **Age-2**  **(#/net night)** |
| --- | --- | --- | --- | --- |
| *Yellow perch* |  |  |  |  |
| 2004 | 171.5 | 0 | 1.3 | 0 |
| 2005 | 5.3 | 3.2 | 0 | 0 |
| 2006 | 211.0 | 16.7 | 16.6 | 0.4 |
| 2007 | 523.1 | 24.7 | 39.3 | 0.3 |
| 2008 | 1154.3 | 8.8 | 17.7 | 0.1 |
| 2009 | 855.8 | 3.7 | 5.0 | 0 |
| 2010 | 433.9 | 4.4 | 1.1 | 0 |
| 2011 | 345.4 | 2.7 | 1.6 | 0 |
| 2012 | 551.1 | 6.8 | 0.2 | NS |
|  |  |  |  |  |
| *Bluegill* |  |  |  |  |
| 2004 | 144.2 | 51.5 | 11.1 | 5.0 |
| 2005 | 923.9 | 44.1 | 0.04 | 0.3 |
| 2006 | 1763.7 | 83.2 | 35.0 | 8.1 |
| 2007 | 732.1 | 8.8 | 0.5 | 1.0 |
| 2008 | 2319.8 | 6.4 | 2.9 | 0.6 |
| 2009 | 428.0 | 209.5 | 6.3 | 2.9 |
| 2010 | 1286.0 | 8.8 | 1.1 | 1.9 |
| 2011 | 1031.0 | 55.8 | 8.3 | 1.1 |
| 2012 | 3218.8 | 15.2 | 0.2 | NS |
